# A technique for repeated blood and cerebrospinal fluid sampling from individual rats over time without the need for repeated anesthesia

**DOI:** 10.1038/s41598-024-55666-6

**Published:** 2024-03-02

**Authors:** Erin Santandrea, Farhang Aliakbari, Emily Truscott, Lynda McCaig, Neil S. Donison, Danielle Graham, Michael J. Strong, Kathryn Volkening

**Affiliations:** 1 Molecular Medicine, Robarts Research Institute, Schulich School of Medicine and Dentistry, University of Western Ontario, London, Canada; 2 Animal Care and Veterinary Services, University of Western Ontario, London, Canada; 3 Clinical Neurological Sciences, Schulich School of Medicine and Dentistry, University of Western Ontario, London, ON Canada

**Keywords:** Cerebrospinal fluid, Cisterna magna, Jugular catheterization, Repeated sampling, Rodent, Exosomes, Biological techniques, Biological models, Neurological models, Diseases, Neurological disorders, Neurodegeneration

## Abstract

Ethical animal use follows the 3R’s: Replacement, Reduction and Refinement. Here, we present the use of simultaneous jugular vein and cisterna magna catheterization via a port system in rats for repeated fluid sampling for 14 consecutive days without loss of catheter patency. This technique allows repeated intra-animal sampling without anesthesia and, if used with pooling samples from a cohort of animals, replaces the need for terminal collections for sufficient sample volumes.

## Introduction

Cerebrospinal fluid (CSF) collection in rodents commonly requires terminal collections^1^ or repeated anesthetics^2^ to obtain sufficient sample volumes. This translates into increased animal use and difficulties in examining disease progression within single animals when time course expression analyses are required, especially in cases where repeated anesthetics can affect the integrity of biological samples^3–6^. We adapted a surgical technique to allow repeated sampling of blood and CSF from the same animal over time without the need for repeated anaesthetics and with minimal restraint. This technique also allows for group housing of animals, reduced restraint requirements, greater ease of handling and less animal stress.

Ongoing work requires the isolation of CSF extracellular vesicles (EVs) from an inducible transgenic rat model of ALS (ChAT-tTA/TRE-TDP-43^M337^^V^ rats^7^) through a time course study. Because the development of a motor phenotype progresses rapidly post-doxycycline withdrawal in these transgenic rats and because the volume of CSF that can be withdrawn is very small (≤ 200 µl/withdrawal), repeated sampling across a cohort of animals over time is desirable.

The use of ports and implantable pumps is common in experiments requiring ongoing dosing or treatment of animals^8–10^. Repeated sampling from animals would be ideal for time-course experimentation where individual differences in animal physiology can affect results. However, this is commonly not possible due to insufficient sample volumes being obtained or because terminal sample collections are needed. Here we employed a system developed for ongoing dosing to withdraw biological samples from animals over time and adapted it to allow for the simultaneous withdrawal of both blood and CSF samples from individual animals for a period of 14 days post-recovery. Catheters remained patent for 17 days.

## Surgical methods

### Animal husbandry

Sprague–Dawley rats (RRID:RGD_737891) were group housed with enrichment in open-air conventional cages and fed LabDiet ProLab IsoPro RMH3000 and powdered Teklad 2018 ad libitum with free access to water. All animals were handled in accordance with CCAC regulations, and all methods were performed in accordance with relevant guidelines and regulations. The study was conducted under an Animal Care Committee approved animal use protocol (#2022-196) at the University of Western Ontario (London, Ontario, Canada). Animals were group housed on a 12 h light/dark cycle.

### Pre-surgical preparation

Rats were anesthetized with 4% isoflurane (Fresenius Kabi Canada Ltd) and maintained on 1.5% isoflurane. Prophylactic saline (10 ml/kg/hr; Pfizer) and 1.0 mg/kg buprenorphine S/R (ACVS, University of Western Ontario) were given subcutaneously. Bupivicain (Aspen Pharmacare Canada Inc., Sensorcaine) was injected subcutaneously between the ears at the cranial surgical site and into the ventral neck surgical site. The surgical sites (base of the skull, between the shoulder blades and the ventral neck) were prepared by clipping and scrubbing with chlorhexidine (Partnar 2% chlorhexidine solution) and surgical scrub (EcoLab Bacti-Stat Antibacterial Hand Soap). The animal was placed on a circulating water heating pad to maintain body temperature and monitored with PhysioSuite (Kent Scientific PS-03). The plane of anesthesia was monitored and adjusted as required.

### Jugular vein catheterization

**Figure 1:**
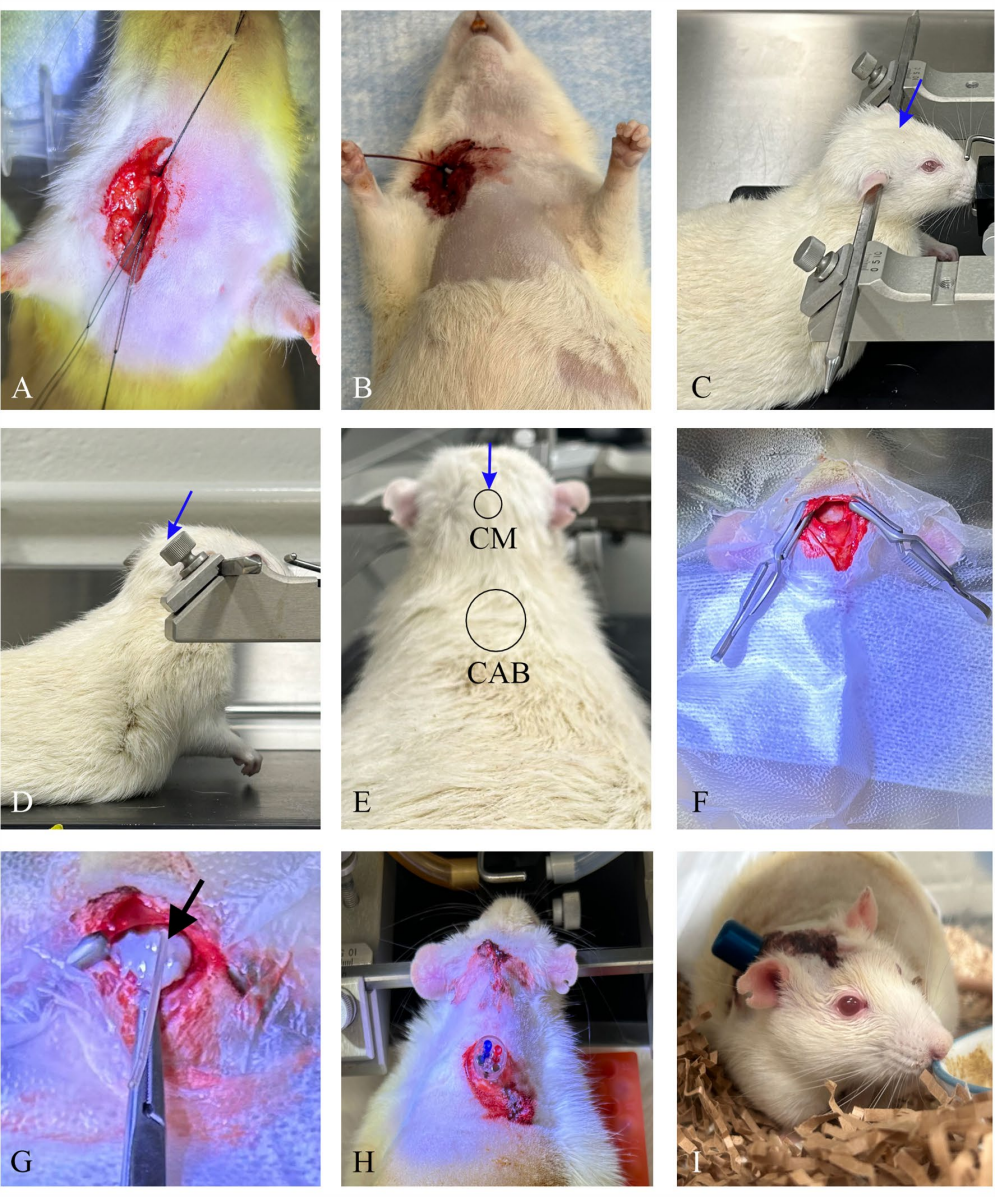
(**A**) Accessing the jugular vein to place the catheter for blood collection. (**B**) Position of the jugular catheter prior to tunnelling to the CAB. (**C**–**E**) Positioning of the rat in the stereotaxic frame to maximize exposure to the cisterna magna for catheter insertion and showing the surgical sites for the cisterna magna (CM) cannula and CAB. A small burr hole is drilled in the location (circle “CM”) and the cannula then gently and slowly advanced into the cisterna magna in the direction of the arrows (blue) in (**C**–**E**). Positioning of the head and neck of the rat to be higher than the body is vital for success in gaining access to the cisterna magna. (**F**) Burr hole in the skull to allow access to the cisterna magna. (**G**) Insertion of the cannula into the cisterna magna (black arrow points to cannula). (**H**) Positioning of the CAB after tunneling the jugular vein and cisterna magna catheters to the CAB. (**I**) Recovered rat post surgery.

A 5 cc syringe was placed under the back of the neck for support while the rat was in dorsal recumbency. The external jugular vein was exposed through a 1 cm incision made approximately 1 cm lateral to the midline (Fig. 1A). One drop of lidocaine (Teligent) was applied to prevent vasoconstriction and the vein isolated with three 4–0 silk ties (Ethicon, Perma-Hand Silk, 683G) at each end of the exposed vessel. The cranial suture was tightened to occlude blood flow and a small incision made in the vessel to allow insertion of a heparinized saline-filled (2 units heparin/ml 0.9% sodium chloride) catheter (SAI Infusions Technologies, Rat Jugular Vein Catheter, Rounded, Beads at 3 cm and 3.5 cm, RCMC-7.6-02). The catheter was advanced past the caudal suture, aspirated to confirm patency, secured by the caudal suture and flushed with heparinized saline. The cranial tie was then used to secure the catheter and the catheter tunneled to the dorsum of the animal after the placement of the dual-channel Catheter Access Button (CAB; below). The incision was then closed with 4-0 monocryl (Ethicon, 4-0 Monocryl Y494G) suture in a subcuticular pattern and tissue glue (3 M Vetbond) (Fig. 1B). The jugular vein catheter was tunneled subcutaneously and externalized in the mid-scapular region, held in place with hemostats for connection to the Catheter Access Button (CAB) after the cisterna magna catheter was placed.

### Surgical catheterization of the cisterna magna and connection to the CAB

After placement and tunnelling of the jugular vein catheter was complete, the animal was positioned into a Kopf stereotaxic frame (Stoelting) with blunt ear bars, with the anaesthetized animal positioned with the body lower than the head. Figure 1C–E show the animal positioning (C, D) and locations for cisterna magna access and CAB position (E). Positioning of the head higher in this manner is vital to successful placement of the cisterna magna cannula. A 2–3 cm midline incision was made through the scalp. The skin and muscle were retracted laterally. Soft tissues were kept moist throughout the procedure. The surface of the skull was etched with a scalpel blade to allow for adherence of dental cement. Using a sterile 1.35 mm stereotaxic drill bit (Stoelting, 514,555), a craniotomy/burr hole was made into the skull over the occipital crest (Fig. 1F), allowing cautious insertion of a 22 g cannula (SAI Infusion Technologies, Rodent CM Cannula, 90 degrees with 7.6 mm tip, SAI RCMC-7.6-02) into the cisterna magna (Fig. 1G). Free flow of CSF from the unplugged catheter indicated correct placement of the catheter. The plug was replaced, and dental cement (Bisco BisCem Self-Adhesive Resin Cement, Dual-Cured, D-45011P) was carefully applied around the cannula and any exposed cranium and allowed to dry completely to secure the cannula in place. The acrylic was smoothed, trimmed, and filed to remove any sharp edges and extra acrylic was cleaned from the skin. Screws were not needed. The cannula was tunneled subcutaneously to the CAB, and the incision closed using 4-0 monocryl and tissue glue.

### Placement of the CAB

The Dual-Channel Catheter Access Button (SAI infusion technologies, Catheter Access Button for Rat 22G, SAI CAB22-R2) was placed between the shoulder blades of the animal through an incision (Fig. 1H) after both catheters were tunnelled and externalized at the CAB location.

### Catheter patency, flushing and post-operative care

Catheter and cannula patencies were confirmed prior to trimming and connection to the CAB. The Cannulock of the CAB was moistened with sterile saline, the catheter/cannula connected and implanted subcutaneously. The CAB was positioned rostrally, and the incision closed in a simple interrupted pattern using 4-0 monocryl and tissue glue (Fig. 1H). Patency was confirmed via CSF and blood withdrawal from the CAB ports. The jugular catheter was locked using 236 µl of catheter locking solution (heparin-glycerol, SAI Infusions, SAI HGS-10). Access ports were covered with magnetic caps (SAI Infusions, CAB-RCR, CAB-BCR or CAB-GCR). The rat was removed from the stereotaxic apparatus and allowed to recover from anesthesia with supplemental heat and vital parameters/reflex monitoring until fully recovered and ambulatory. Rats were monitored postoperatively three times daily for four days and then at least once daily until endpoint (Fig. 1I). The animals were cohoused upon recovery from anesthesia. Additional subcutaneous saline and/or Buprenorphine S/R were provided if necessary. Withdrawal of CSF can lead to changes in intracranial pressure leading to pain and altered mental state. Careful observation of behaviour and support with hydration to allow for replenishment of CSF and pain control is essential should the rat’s behaviour shows any evidence of distress. Blood and CSF collections were started 72 h after the CAB placement and continued daily to 14 days.

### Blood collection

Each rat was weighed and the volume of blood to be collected was calculated as: 0.75% of the animal’s body weight in grams = weekly limit of blood collection, with 1 g blood being equivalent to 1 g weight^11^. The rats were wrapped in a towel for restraint, the magnetic cap removed from the CAB, and the ports accessed using a syringe fitted with a blunt tipped adaptor (SAI Infusions, SAI CLPAD-100). Using aseptic techniques, twice the volume of the catheter was withdrawn and discarded. The sample collected into an EDTA-blood collection tube. The catheter was flushed with 4 times the catheter volume (945 µl) with heparinized saline and then locked with 236 µl locking solution with positive pressure to prevent any blood clotting at the end of the catheter.

### CSF collection

Rats have a total CSF volume of 200–275 µl and CSF is produced at 3.7 µl/min^10^. Using syringes fitted with the catheter access port adaptor as above, first the volume of the cannula was collected (~ 15 µl), then the sample of CSF collected (50–150 µl volume) and the catheter flushed with 15 µl of PBS. Animals were examined daily for signs of pain, discomfort and for normal behaviours. For any animals that did display pain, treatment with buprenorphine and additional hydration through saline injection was advised, although no animals showed evidence of pain or discomfort over the duration of this study.

### Euthanasia

The endpoint for these animals was 14 days post recovery (17 days from CAB placement). Anesthetic overdose was used.

## Confirming CSF sample quality methodology

### Isolation of EVs from blood and CSF samples

Blood collected into EDTA tubes was spun at 3000 rpm for 10 min to remove cells, and the plasma saved for EV extraction. Blood samples were then pooled to obtain > 10 ml of blood. CSF samples were pooled from individual animals to obtain 500 µl total volume. EVs were isolated using ultracentrifugation at 100,000 g for 120 min at 4 °C in polycarbonate tubes (Beckmann, 8 × 51 mm #355,657). The supernatant was removed from the tube, and the pellet was resuspended in 100 µl cold phosphate buffered saline (PBS pH 7.2; Invitrogen) containing cOmplete proteinase inhibitor (Roche; 1 tablet/50 ml PBS) and RNAseOUT (Invitrogen; 5 µl/50 ml PBS).

### Western blotting for exosomal markers

EV fractions isolated by ultracentrifugation were loaded onto 12% SDS-PAGE gels, transferred to nitrocellulose membrane, blocked for 1 h at room temperature in TBS + 0.1% Tween 20 containing 10% skim milk powder and probed for exosomal markers with RαAlix (Proteintech, #12422-1-AP; RRID:AB_2162467), RαCD81 (Abcam, #ab109201; RRID:AB_10866464), RαHsp90 (Proteintech, #13171-1-AP; RRID:AB_212-924), MαSyntenin-1 (Santa Cruz, #sc-515538), or RαTSG101 (GeneTex, #GTX64349) overnight at 4 °C in blocking buffer. Membranes were washed in TBS + 0.1%Tween 20, and probed with secondary antibodies (HRP-conjugated GαM (BioRad, #1706516; RRID:AB_11125547) or HRP-conjugated GαR (Invitrogen, #65-6120; RRID:AB_2533967)) for 1 h at room temperature. The blots were washed, and then signal developed with the Western Lightning Plus ECL reagent (FroggaBio, #NEL104001EA) and visualized with a BioRad Gel Documentation Centre. When needed, membranes were stripped with ReStore Stripping Buffer (ThermoFisher, #PI21059).

## Results and discussion

We used the rat catheter access button (CAB) system from SAI Infusion Technologies (Lake Villa, IL, USA) to place catheters into the cisterna magna and jugular vein, which were then tunnelled to and connected to the CAB for easy access. The CAB ports (pin ports) were secured from contamination by a magnetic port cover. The use of a small adapter allows access with any appropriately sized syringe. All ventrally located surgery was completed first so that the animal was absolutely immobile during the insertion of the cannula into the cisterna magna. The jugular vein was catheterized first and tunnelled to the CAB position prior to insertion of the cisterna magna catheter and the CAB.

Orienting the animal into a stereotaxic frame with the head held higher than the body was critical to gaining access to the cisterna magna (Fig. 1C, D). This prevented damage to the cerebellar tissues by utilizing gravity to pull structures down and thus opening a small access for implanting the catheter (Fig. 1G). The catheter must be advanced slowly and care must be taken to properly cement the catheter firmly in place prior to tunneling to the CAB. Failure to use dental cement in this stage will result in the dislodging of the cannula and possible damage to the cerebellum resulting in severe distress for the animal.

**Figure 2:**
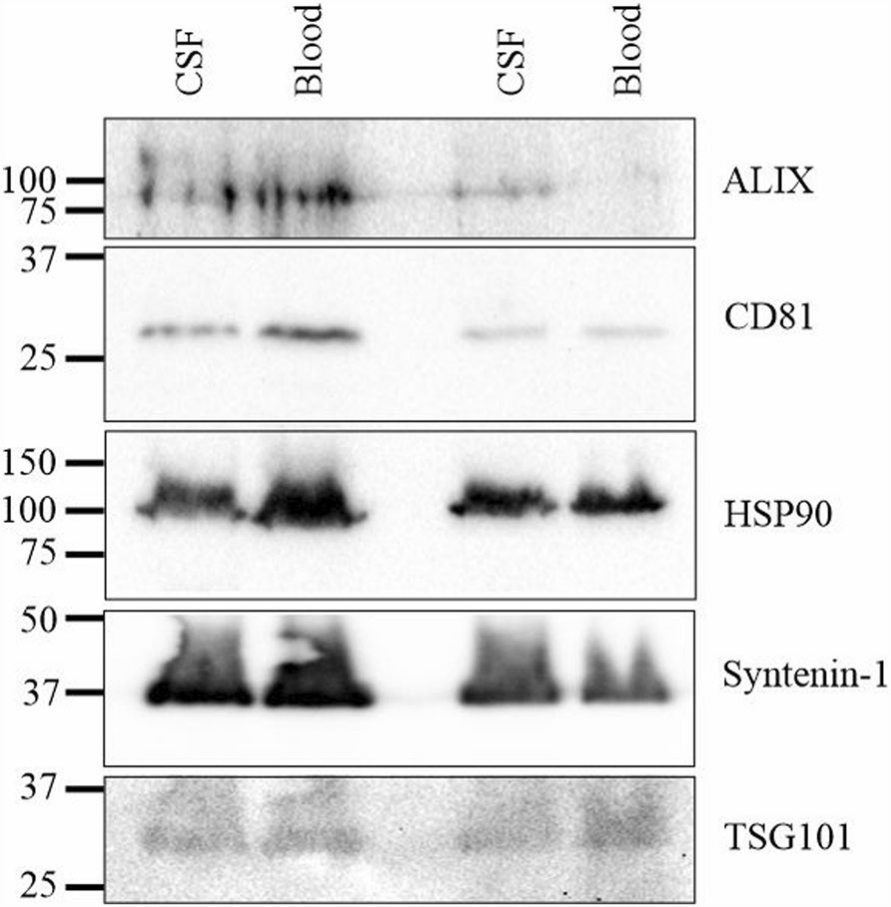
Representative western blots of exosomes isolated from cerebrospinal fluid (CSF) or blood from 2 different animals probed for common exosomal markers: ALIX (95 kDa); CD81 (26 kDa); HSP90 (90 kDa); Syntenin-1 (35 kDa) and TSG101 (32 kDa). Molecular weight ladders are denoted in kDa to the left of each blot. See Suppl Fig. 1 for full western blots.

Even though this is a highly technical surgical technique, it allows daily sampling from rats with minimal restraint, for example, simply wrapping them with a towel. Daily sampling easily allows an animal to be used as its own control (prior to treatment) and helps minimize data deviations caused by using populations of individual rats. To determine if time course experiments following the onset of disease could be performed with this technique, we collected samples daily from 4 individual animals for up to 14 days postsurgery for EV isolation. We obtained 200 µl blood/day/animal and 50 µl of CSF/day/animal for 3 animals and 150 µl of CSF/day for 1 animal (Table 1). CSF and blood samples were subjected to ultracentrifugation to isolate EVs. EV isolates were subsequently analyzed by western blotting for common EV markers, including ALIX (ALG-2 interacting protein X), CD81 (tetraspanin-28), HSP90 (heat shock protein 90 kDa), Syntenin-1 and TSG101 (tumor susceptibility 101) (Fig. 2; see Suppl Fig. 1 for full uncropped gels). We observed that the collection of CSF and blood samples using this method can yield samples that can be pooled successfully for EVs isolation.

**Table 1:** Blood and CSF collections per animal per day post surgery. All volumes are presented in microliters. Days 1–3 no samples were collected to allow for surgical recovery. All volumes are reported in microliters.

Animal	Rat 1		Rat 2		Rat 3		Rat 4	
Day	Blood	CSF	Blood	CSF	Blood	CSF	Blood	CSF
1	–	–	–	–	–	–	–	–
2	–	–	–	–	–	–	–	–
3	–	–	–	–	–	–	–	–
4	200	50	200	50	200	150	200	50
5			200	50	200	150	200	50
6			200	50	200	150	200	50
7			200	50	200	150	200	50
8			200	50	200	150	200	50
9			200	50	200	150	200	50
10					200	150	200	50
11					200	150	200	50
12					200	150	200	50
13					200	150	200	50
14					200	150	200	50
Total	200	50	1200	300	2200	1650	2200	550

In summary, we have adapted a technique usually used for repeated dosing and treatment delivery in rats for repeated, concurrent sampling of CSF and blood. The catheters remained patent for 14 days post recovery, the end point of this proof-of-principle experiment. With proper care, flushing and locking, these catheters should remain patent much longer than the period that was trialed here. By using this surgical technique, the numbers of animals required for time-course study can be decreased (elimination of terminal sample collections), each animal can serve as their own controls, and animals are free to be group housed, can engage in their normal daily activities, and not need to undergo repeated anesthesia for sample collection.

### Supplementary Information

Supplementary Figures.

## Data Availability

All data generated and analyzed during this study are included in this article.

## References

[1] Sharma AK (2006). Development of a percutaneous cerebrospinal fluid collection technique in F-344 rats and evaluation of cell counts and total protein concentrations. Toxicol. Pathol..

[2] Liu L, Duff K (2008). A technique for serial collection of cerebrospinal fluid from the cisterna magna in mouse. J. Vis. Exp..

[3] Piao L (2022). Effects of general anaesthesia with an inhalational anaesthetic agent on the expression of exosomes in rats. Int. J. Med. Sci..

[4] Altholtz LY, Fowler KA, Badura LL, Kovacs MS (2006). Comparison of the stress response in rats to repeated isoflurane or CO_2_:O_2_ anesthesia used for restraint during serial blood collection via the jugular vein. J. Am. Assoc. Lab. Anim. Sci..

[5] Upton DH, Popovic K, Fulton R, Kassiou M (2020). Anaesthetic-dependent changes in gene expression following acute and chronic exposure in the rodent brain. Sci. Rep..

[6] Colon E (2017). Anesthesia, brain changes, and behavior: Insights from neural systems biology. Prog. Neurobiol..

[7] Huang C, Tong J, Bi F, Zhou H, Xia XG (2012). Mutant TDP-43 in motor neurons promotes the onset and progression of ALS in rats. J. Clin. Invest.

[8] Nolan TE, Klein HJ (2002). Methods in vascular infusion biotechnology in research with rodents. ILAR. J..

[9] Glud AN (2019). Visualization of intrathecal delivery by PET-imaging. J. Neurosci. Methods.

[10] Prelusky DB, Hartin KE (1991). A technique for serial sampling of cerebrospinal fluid from conscious swine and sheep. Lab. Animal Sci..

[11] Canadian Council on Animal Care. Canadian Council on Animal Care guidelines: Rats. (2020). http://www.ccac.ca

