# Supplementary material for: A technique for repeated blood and cerebrospinal fluid sampling from individual rats over time without the need for repeated anesthesia
Source: Sci Rep. 2024 Mar 2;14:5171. doi: 10.1038/s41598-024-55666-6 (PMC10908789; doi:10.1038/s41598-024-55666-6)
Supplement: Supplementary file 1 — Supplementary Figures. [file 41598_2024_55666_MOESM1_ESM.pdf]

A

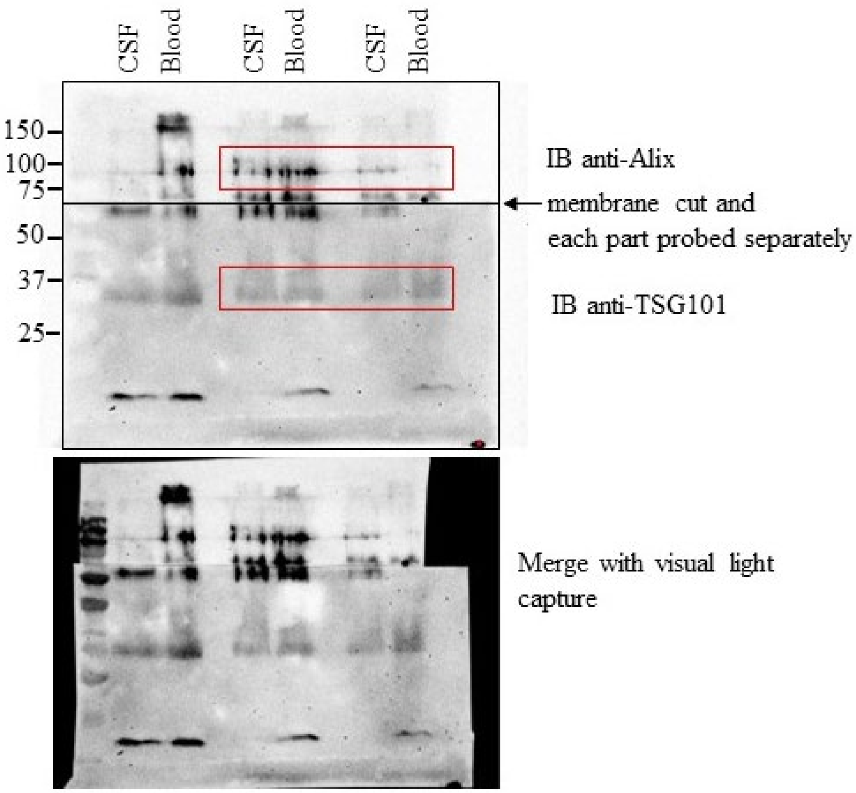

Supplemental Figure 1A: Alix and TGS101 full western blots are shown as just the chemiluminescence capture (upper panel) as well as combining with visual capture (bottom panel) using an automated Biorad Gel Documentation Center. The membrane was cut after transferring and prior to blocking so that each section could be probed with a different antibody. Red rectangles outline areas shown in Fig 1.

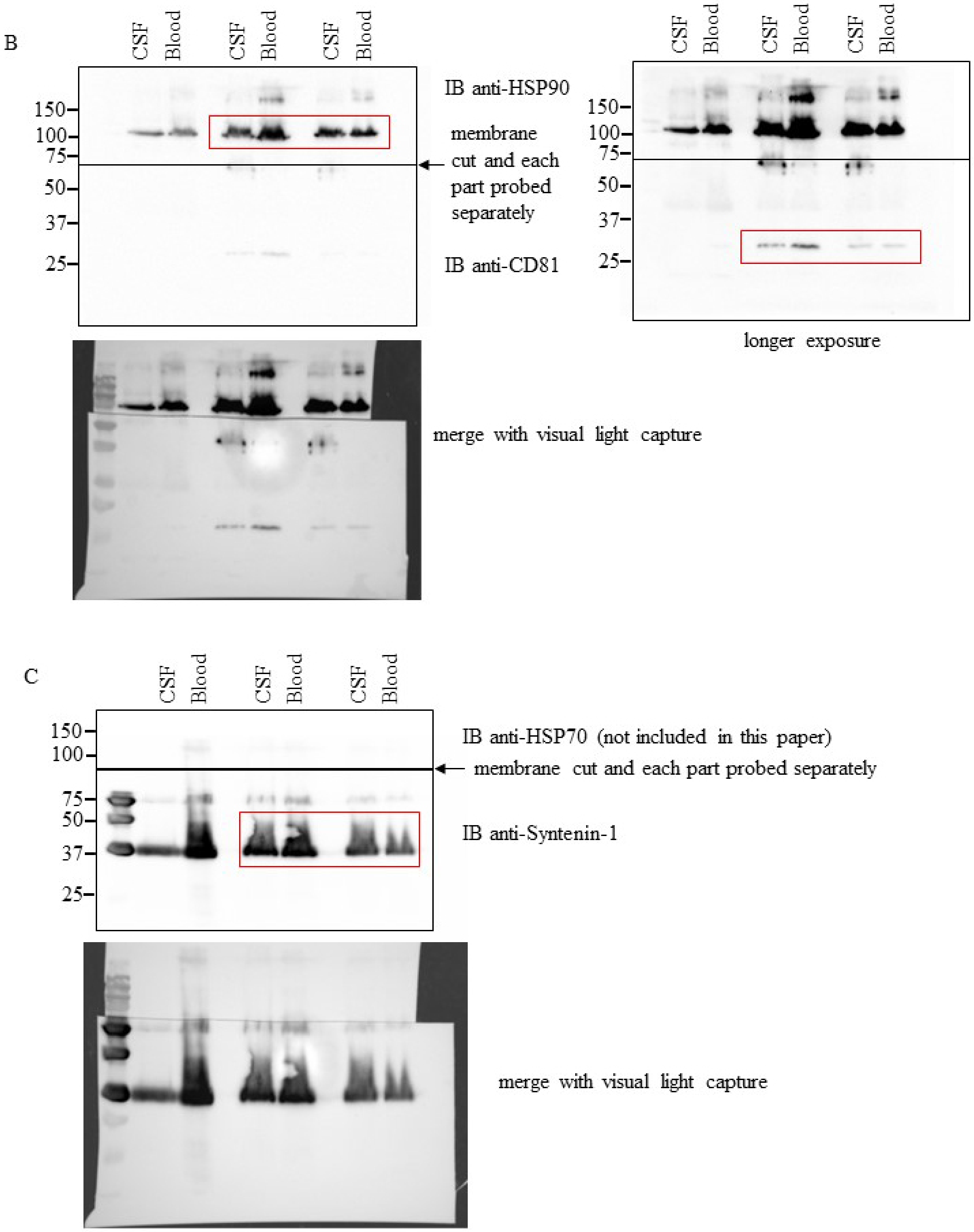

Supplemental Figure 1B and 1C: Full western blots are shown as just the chemiluminescence capture (upper panels) as well as combining with visual capture (bottom panels) using an automated Biorad Gel Documentation Center. Each membrane was cut after transferring, and

prior to blocking so that each section could be probed with a different antibody. Red rectangles outline areas shown in Fig 1. B: HSP90 and CD81; C: HSP70 (not shown in Figure 1) and Syntenin-1.
